# Immunophenotyping of Non-Hodgkin's lymphomas in Sudan

**DOI:** 10.11604/pamj.2014.18.82.3732

**Published:** 2014-05-24

**Authors:** Khalid Hamad Hamid, Adel Hussein Elduma, Badereldin Mirgani Yousif Mohamed, Magdi Mansour Abdel Farage Salih

**Affiliations:** 1 National Public Health Laboratory, Khartoum, Sudan; 2 University of Khartoum, Sudan

**Keywords:** Immunophenotyping, Non-Hodgkin's lymphoma, lymphoproliferative disorders, Sudan

## Abstract

**Introduction:**

Non-Hodgkin′s lymphomas (NHLs) are heterogeneous group of malignant lymphoproliferative disorders.

**Study objectives:**

This was a retrospective study aimed to classify NHLs into B cell and T cell types; in addition to demonstrate the histological patterns and correlate it with gender, age and site of the biopsy.

**Methods:**

The study was conducted in Histopathology Department, National Heath Laboratory, during the period 2007-2010. Formalin fixed paraffin wax embedded tissue blocks which were diagnosed as NHLs by routine Haematoxylin and Eosin (H&E) stain during the period 2000-2008 were used. Haematoxylin and Eosin (H&E) stain were done. Immunohistochemistry stains performed according to Dako cytomation protocol 2007. Lymphoid markers which were used in this study are CD45 (LCA), CD20 (B cell marker), CD3 (T cell marker), CD15 and CD 30. Epithelial marker which was used is CK MNF116. The total number of samples collected was 66; two of them were excluded because of poor processing. Another two specimens were excluded because they are non-reactive with lymphoid markers. The remaining 62 specimens were confirmed to be NHLs and classified into B cell and T cell types.

**Results:**

The study showed that B cell NHLs represented 87.1% while T cell NHLs were 12.9%. The Male: Female ratio was 1.6:1. The major affected age group was (47-67) years (38.1% of all specimens). The most frequent histological grade was intermediate grade NHLs (27% of all specimens). The most common site of NHLs in this study is the lymph node (40% of all specimens) followed by stomach (19.4%).

**Conclusion:**

Extranodal locations are the most common sites affected with T cell NHLs. In conclusion; this study confirmed the fundamental role of immunohistochemistry in diagnosis and classification of NHLs.

## Introduction

Malignant lymphoma is a primary malignant neoplasm of lymphoid tissue arising from the expansion of malignantly transformed lymphocytes, which may contain one or more genetic abnormalities [1]. It is divided into two broad categories; Hodgkin's lymphoma and Non-Hodgkin's lymphomas (NHLs). Genetic alternations, viruses and environmental agents as well as radiotherapy and chemotherapy are implicated as etiologic factors [2]. Non-Hodgkin′s lymphomas (NHLs) constitute heterogeneous group of malignant lymphoproliferative disorders. It can arise from nodal or extra nodal locations and spread in unpredictable fashion. Two thirds of NHLs and virtually all cases of Hodgkin′s lymphomas present with non-tender nodal enlargement (often greater than 2 cm). The lymphadenopathy can be localized or generalized. The remaining one third of NHLs arises at extra nodal sites such as skin, stomach and brain. The extra nodal location found in approximately 20% of patients with limited stage high grade disease [3]. This study used routine Haematoxylin & Eosin (H&E) and immunohistochemical stains in tissue sections obtained from formalin fixed paraffin wax embedded tissue blocks previously diagnosed as Non-Hodgkin′s lymphomas. The immunohistochemical markers which were used for confirmation and classification of NHLs include Leucocyte Common Antigen (LCA), CD20 (B cell marker), CD3 (T cell markers), CK (epithelial marker), CD30 and CD15 (Reed-Sternberg cell markers).

The etiologic factors of NHLs included genetic alterations, weak immune system, certain drugs after an organ transplant, and certain infectious agents such as Helicobacter pylori, HIV, Human T-cell leukemia/lymphoma virus type 1, Hepatitis C virus and Epstein-Barr virus (EBV) [2, 3]. Survivors of NHLs have an increased risk of second malignancy. One study suggests that the secondary head and neck cancer can be regarded as one of the late complications of radiotherapy for NHLs of the head and neck [4]. Increased incidence of NHLs has been reported among farmers and those who work with pesticides [5].

In Sudan, little work has been done concerning NHLs. In Soba Teaching Hospital during the period 1979-1989 they found that there were 1205 patients with malignancy, 51 patients of them with NHLs (comprising 5.4% of all malignant tumors). The male-female ratio was 4.1:1, the age of patients ranged between few months to 90 years old, and the age group (40-70) years show higher frequency of NHLs [6].

The incidence of extra nodal NHLs is increasing worldwide. The evaluation of cases of NHLs in Kuwait between 1998 and 2003 according to the site of presentation and their classification in to primary nodal and extra nodal revealed that there were 422 cases of NHLs diagnosed at this period, among which 97 cases (23%) were primary nodal, 132 cases (31%) were early nodal, and 193 (46%) were disseminated primary nodal. In general, there was a male prevalence of primary nodal cases (63%). The most common histological subtype among extra nodal cases was diffuse large cell lymphoma which accounted 71%. The most common anatomic site involved was gastrointestinal tract, which accounted 45% of all cases [7].

T cell non-Hodgkin′s lymphoma represents approximately 10-15% of all lymphomas diagnosed in western countries. Varied geographic frequency of T cell NHLs has been documented ranging from 18.3% of NHLs in Hong Kong to 1.5% of NHLs in Vancouver. This may in part be a reflection of increased exposure to pathologic factors such as Human T cell leukemia virus-1 and Epstein-Barr virus in Asian nations. T cell NHLs commonly present with extra nodal sites [8]. The incidence of T cell NHLs is higher in Far East Asia than in Western countries. A study conducted in 586 Korean patients with NHLs, 101 (17.2%) were T cell NHLs with the most frequent subtype extra nodal NK/ T cell NHLs [9].

In Pakistan a retrospective study reviewed 557 cases of diffuse large B cell NHLs from 1988 to 2004. The median age was 48.7 + /-15.3 years, M: F ratio was 2:1. The distribution according to the primary site was lymph node 322 cases (58%), extra nodal site 325 cases (42%) including gastrointestinal tract 63%, bones 8%, spine 5%, and unusual sites less than 3% [10]. In Greece among 810 cases with NHLs there were 435 males and 375 females. 95% of them aged 11].

In southeast Turkey retrospective analysis carried out on 490 cases of NHLs, with the purpose of evaluating the clinico-pathologic features of these patients. The patients were assessed with regard to their characteristics including age, gender, and histological distribution, stage, extra nodal involvement, presenting symptoms, and biopsied site. Of the patients 314 (64%) were males and 176 (36%) were females. The overall median age was 43 years (ranged: 14-90 years). The patients were classified according to the Working Formulation (WF) system: 71 (14.4%) were low grade, 342 (69.8%) were intermediate grade, 43 (8.7%) were high grade, 34 (6.7%) had other lymphomas. 320 cases of intermediate grade NHL reveled that 78% were B cell lymphoma, whereas 16% were T cell lymphoma and 6% were unclassified lymphoma. Extra nodal involvement was found in 218 (44.5%) patients. The most common affected sites were small bowel, stomach, and tonsil 72 (33%), 63 (29%), and 19 (8.7%) respectively [12].

Fifty three cases of NHLs were studied in India. Of these, seven cases with primary extra nodal lymphomas. A detailed morphological assessment was done and classified using the international working formulation. The two most common types encountered were diffuse large cells lymphoma and small lymphocytic lymphoma. Immunohistochemistry was done and revealed thirty eight (72%) showed B cell expression and 12 cases (22.5%) showed T cells expression. Three cases did not express either marker. B cell diffuse large cell lymphoma (26%) was found to be predominant B cell NHL. The commonest T cell lymphoma was T cell lymphoblastic lymphoma (67%) [13].

NHLs are common neoplasm in Middle East. The extra nodal forms are apparently far more frequent there than in the west. In addition, a peculiar form of primary intestinal lymphoma, namely proliferative small intestinal disease, has been described in Middle Eastern and Mediterranean countries. Several reports indicate high incidence of NHLs in Turkey, for 4 years 185 patients with NHL were in Cukurova University hospital. This constituted 13% of all malignant neoplasm diagnosed in oncology clinic, the mean age for men was 45.5years and 41 years for women. 54% of these cases were nodal lymphoma and the remaining 46% were extra nodal lymphoma, the stomach was the most common localization (43%) followed by the intestinal involvement (30%) and the abdominal mass (27%) [14].

In Japan Waldyey-er's ring lymphomas is common and comprises about 10-20% of all lymphomas. In contrast, in USA the frequency of involvement of Waldyey-er's ring lymphoma has been reported to be about 7-10%. It should be included in nodal or extra- nodal lymphomas. It will be very difficult to conclude the argument now; however, it is quite clear that W-NHL has unique clinical features different from those other nodal or extra-nodal lymphoma.

One hundred and four unselected cases of NHLs in adult Chinese patients in Hong Kong were typed, using monoclonal and conventional antibodies, by immuno-enzymetic labeling methods on cryostat sections or cell smears. The total included 69 cases (66%) of B cell and 26 cases (25%) of T-cell. The diffuse large cell (centroblastic or immunoblastic) types formed the largest proportion 45% of B cell NHLs. Of 26 cases of T cell lymphoma 25 were peripheral type; of these 25, the most frequent subtype (42.3%) is immunoblastic lymphadenopathy-like lesion. The incidence of T cell lymphoma in china is not markedly higher than that of western countries [15]. Primary malignant lymphoma of central nervous system (CNS) is an uncommon tumor, which account of approximately 0.5% of primary brain neoplasm, and less than 2% of extra nodal lymphomas. There is increased frequency in patient with hereditary and acquired immune deficiency states, including those who have received prolonged immune suppressive medication or after organ transplantation. Demonstration of Epstein–Barr viral genome in primary brain lymphoma has resulted in speculation about a causal relationship [16]. The average of annual United States incidence of brain lymphoma is 5.4 cases per ten million populations. The incidence of this tumor is slightly higher among men than women, and the average age at onset is 57 to 59 years range (9 to80) years of age. There is increase in incidence of this primary brain NHL in immunological normal person unrelated to acquire immunediffiency syndrome (AIDS) and organ transplant [17]. Although mediastinal involvement is at presentation in 15% to 25% of patient with NHLs, it is more frequent during the evaluation of the disease in association with nodal and or extra-nodal involvements. Pure mediastinal lymphomas are rare. The most common is lymphoblastic lymphoma and they display an immunologic-phenotype and have some peculiarities that distinguish them from acute lymphoblast leukemia [18]. Pediatric NHL comprises an important subset of childhood malignancies with characteristic clinical, histologic, cytogentic, and immunologic findings. Lymphoblastic lymphoma, small non-cleaved cell lymphoma and large cell lymphomas comprise the vast majority of child hood NHLs. Proper distinction of this subset of NHLs has important therapeutic and prognostic implication for the patients [19]. Colon is infrequently involved as a primary location of NHLs accounting for 4% of all extra nodal NHL and far less than 1% of all colonic malignancy. Colonic NHLs differs significantly in terms of presentation, therapy and outcome relative to other more common gastrointestinal sites, such as stomach or small bowel. The most common location is the cecum (60-74% of cases).

A retrospective study was conducted on extra nodal NHLs diagnosed at the surgical pathology laboratory at Buenos Aires University, in Argentina between1985 and 2004. Overall mean age of the patient was 49.4 and the frequency was greater in males. All cases were B cell phenotype with a higher frequency of high grade lymphomas [20].

A histological study of adult lymphoma was conducted in Lagos aimed to document the histologic types, age and sex distribution. The male and female accounted for 64% and 36% giving M: F ratio1.8:1. The most frequent site was cervical lymph node. The age group (46-55) accounted about 65% of patient of NHLs. Intermediate grade, high grade and low grade variants of NHL accounted for 39%, 34%, and 27% [21].

There is a wide variation in the prevalence of various subtypes of NHLs worldwide. During the period 1998 to 2006 the prevalence of different subtypes of NHLs were determined based on age, sex, site of disease and ethnicity in Kuwait. The ethnicity group comprised Kuwaiti Arab, non Kuwaiti Arab, Asians and others. The prevalence of B and T cell NHL was 81.8% and 14.2%, retrospectively. The most common age group was (41-60) years old. The most common subtypes in Kuwaiti Arabs were diffuse large B cell lymphoma (45.5%), follicular lymphoma (15.5%) and mycosis fungioides (9.3%). In non Kuwaiti Arabs, the most common subtypes were diffuse large B cell lymphoma (48%), B cell small lymphocytic lymphoma (15.8%) and follicular lymphoma (12.7%). Overall non Kuwaiti Arabs exhibited the highest prevalence (59%) and 54% cases had extra nodal presentation [22].

Bone morrow biopsy is an integral part of staging work-up for non-Hodgkin′s lymphomas. Characteristic of bone morrow involvement in NHLs with respect to incidence, histological pattern and morphology of infiltration and its discordance with the history of primary anatomic site revealed that the involvement of bone morrow by lymphoma showed to be 27 cases 55.1%. The incidence of involvement was higher in T cell when compared with B cell NHL [23].

Extra-nodal NHLs preferentially involve the skin. Among them, 65% are T cell NHLs, 25% B cell. Mycosis fungoides is the most common form of low grade malignant peripheral cutaneous T cell lymphomas. Lymphomas originated from follicular center cells are the most common type of cutaneous B cell lymphoma. The prognosis is good, since the average survival time from diagnosis is 12-14 years. Primary malignant lymphoma of skin accounted about 1-3.5% of all NHLs in children. It has been suggested that these lymphomas represent a unique entity occurring in younger age than usually expected in children with NHLs [24].

Primary cardiac lymphoma is defined as NHL mainly located in heart and/or the pericardium. It is rare and affects the elderly men. Common manifestations are pericardial effusion and heart failure. The diagnosis is usually late and prognosis is poor. This study aim to Classify NHLs by using immunohistochemistry into B-cell and T-cell, demonstrate histological patterns of NHLs and to correlate histopathology of NHLs with age, gender and the site of biopsy.

## Methods

**Study design:** This is a retrospective study aimed to review cases reported as Non–Hodgkin′s lymphomas by routine histopathology stain Haematoxylin &Eosin (H&E). The diagnosis was confirmed and classified them into B-cell and T-cell NHLs by using Immunohistochemistry

**Study area:** The study was conducted in department of histopathology in National Heath Laboratory in Khartoum during the period 2007-2010. In this period of time the national health laboratory received specimens from the entire parts of Sudan

**Study sample:** Formalin fixed, paraffin wax embedded tissue blocks from lymph node or extra nodal locations were used.

**Sample size:** All samples reported as Non-Hodgkin′s lymphoma during the period 2000-2008 were included in the study. The total number of sample was 66 samples.

### Laboratory procedures

From each formalin fixed paraffin wax embedded block three microns sections were cut by rotary microtome and then placed on slide which was labeled by laboratory number of the block; for routine histological stain(Haematoxylin& Eosin) as described by Mayer, 1903, clean, grease-free slides were used while commercial silanized slides provide by(Dako Cytomation 2007), with labeled of marker were used for immunohistochemistry stain. Both type of slides incubated in oven at 60C for 1 hour. Take sections to Distilled water: placed them in Xylene (5mins) 2 changes to remove wax hydrated through absolute ethyl alcohol (5mins) and then 90% ethanol alcohol (5mins) and then 70% ethanol alcohol (5mins) and placed in Distilled Water (D.W).

**Data analysis**: The data obtained from this study analyzed and interpreted by SPSS

## Results

**Figure 1:**
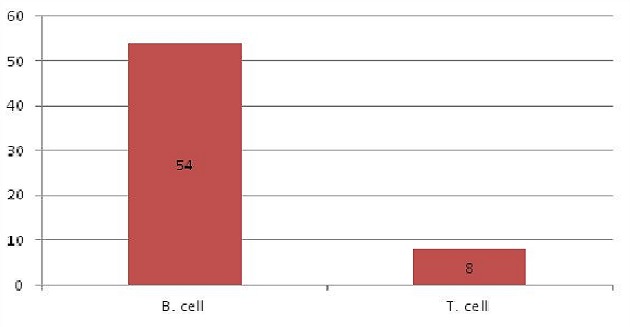
Immunopheotyping of NHLs into B and T cell

**Figure 2:**
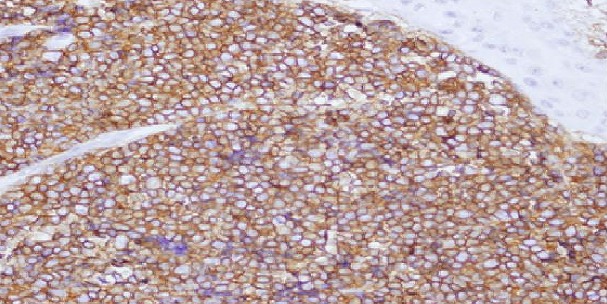
Breast tissue section positive for B cell marker (X10)

**Figure 3:**
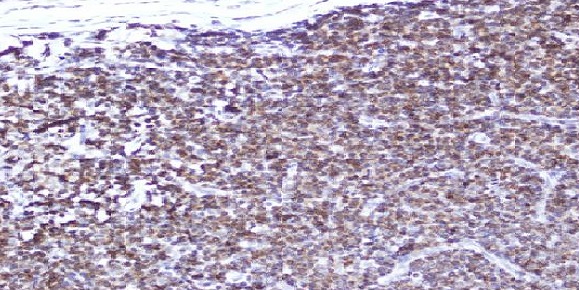
Occipital lymph node positive for T cell marker (X10)

Sixty six paraffin blocks were collected retrospectively from archive of Histopathology Department in National Health Laboratory. These specimens were previously diagnosed as Non-Hodgkin's Lymphomas (NHLs) using routine H&E stain. Two samples were excluded for their inadequate processing which were not suitable for performing immunohistochemistry stain. After use of immunohistochemistry another two samples were excluded because of their positivity for Cytokeratin (epithelial cell marker) and their non-reactivity for lymphoid marker LCA (Leukocyte Common Antigen). The study was performed in 62 specimens. The specimens were reviewed and the diagnosis were confirmed as NHLs then classified into B cell and T cell types using immunohistochemistry. Both sexes were included in this study, males constituted 38 cases (61.3%) while females were 24 cases (38.7%). Male: female ratio is 1.6:1. The ages of the study ranged from 5 years to 87 years. The mean age was 47 years. The age group (47-67) years showed higher incidence of NHLs, comprising 24 cases (38.7%). The age group (26-46) years showed the lowest incidence of NHLs, comprising 8 cases (12.9%). The sites of biopsies were as follow 25 cases (40.3%) lymph nodes followed by 12 cases (19.4%) stomach; while other sites of biopsy were 25 cases (40.3%). The most frequent type of lymph node in this study was the cervical lymph node which represents 9 cases (14.5%) of all site of specimens (Table 1). The other sites of biopsies revealed that the intestinal masses were constituted 5 cases (8%) of biopsy. The histological pattern of NHLs was graded into high grade, intermediate grade, low grade and others. The most frequent grade was intermediate grade NHLs which account 17 cases (27%). Immunoperoxidase technique was performed and revealed that 62 cases were positive for lymphoid marker Leucocytes Common Antigen (LCA), which were sub-divided into 54 cases (87.1%) reactive for CD20 (B cell marker) and 8 cases (12.9%) reactive for CD3 (T cell marker) (Figure 1, Figure 2, Figure 3). The association between NHLs and age showed that the age group (47-67) years representing higher incidence of B cell NHLs (23 cases), while the age group (26-46) years had lowest incidence of B cell NHLs (6 cases). The age groups (5-25) years and (47-67) years show the highest incidence of T cell NHL (Table 2) The relation between histological grade and age was that the age group (47-67) years showed eight cases the higher frequency of intermediate grade NHL while the age group (5-25) years show the higher frequency of high grade NHL six cases (Table 3) The distribution of B cell and T cell NHLs according to gender showed that 21 cases were female affected with B cell NHLs, while three cases were found to be having T cell NHLs. The study showed that 33 cases were males with B cell NHLs and 5 cases were males with T cell NHLs. Concerning the site of the biopsy and its association with B cell and T cell NHLs; the lymph nodes were accounted 22 cases with B cell and three cases with T cell NHLs, followed by stomach which were affected with 10 cases B cell NHLs and two cases with T cell NHL while all other sites of biopsy reported with 22 cases B cell NHLs and three cases T cell NHLs.

**Table 1:** Distribution of lymph nodes

Lymph node	Frequency	Percentage
Inguinal	6	9.6%
Axillary	4	6.5%
Cervical	9	14.5%
Mesenteric	3	4.8%
Unknown site	3	4.8%
**Total**	25	40.2%

**Table 2:** Age groups with Immunopheotyping and site of biopsy

Age	Immunophenotyping				
	B cell	T cell	Lymph node	Stomach	Other site of biopsy
5 – 25	14	3	6	-	10
26 -46	6	-	1	1	4
47 -67	23	3	16	5	4
68 – 87	11	2	2	6	7
**Total**	54	8	25	12	25

**Table 3:** Histological pattern with age and gender

Histological patterns	Age	Male	female	Total
**High grade**	5 - 25	4	2	6
	47 -67	4	1	5
	68 - 87	4	1	5
	Total	12	4	16
**Low grade**	47 -67	2	2	4
	Total	2	2	4
**Intermediate grade**	5 - 25	2	1	3
	26 -46	2	2	4
	47 -67	4	6	10
	Total	8	9	17
**rkitt's type**	5 - 25	1	1	2
	Total	1	1	2
**Diffuse large cell**	5 - 25	2	1	3
	26 -46	2		2
	47 -67		3	3
	68 - 87	5	1	6
	Total	9	5	14
**Small cell**	47 -67	1	-	1
	Total	1	-	1
**Anaplastic large cell**	47 -67	1	-	1
	Total	1	-	1
**Unclassified**	5 - 25	1	2	3
	47 -67	3	1	4
	Total	4	3	7

The distribution of histological patterns into B cell and T cell NHLs revealed higher incidence of B cell in all histological patterns compared to T cell NHLs.

## Discussion

Non-Hodgkin′s lymphomas (NHLs) are heterogeneous group of malignant lymphoproliferative disorders. This study showed that B cell NHLs represent 87.1% while T cell NHLs were 12.9%. This high percentage of B cell NHLs was long-established by Ameen R et al. Their results showed that B cell NHLs were 81.8% and T cell NHLs were 14.2% [22]. These findings were also determined by H O FC et al in china and Kaylan K et al whom they found 66% and 72% of B cell NHLs respectively [13, 15]. Unexplained highest incidence of B cell NHLs were reported by Keszler A et al whom they found 100% of cases were B cell NHLs [20].

Andrew M et al found in Hong Kong 1.5% of patient with NHLs were T cell type. This low incidence of T cell NHLs may be due to the reported varied geographic frequency of T cell NHLs in China [8]. The study of histological grading of NHLs revealed highest frequency of intermediate grade (27%). This was agreed with Anunbi CC et al whom they found (39%) of cases was intermediate grade NHLs [21]. Economophos et al as well found that 66.7% of cases were intermediate grade [11]. Abdurrahman Isikogan et al likewise support our findings because they found a highest frequency of intermediate grade NHLs (69.8% of cases). Keszeler A et al found that the most frequent grade was high grade NHLs [12]. The distribution of T cell NHLs according to the site of biopsy revealed that most common site of biopsies was extra nodal sites 1.7: 1. These findings were decided with Ko OB et al and Andrew M et al whom they noticed that the most common site for T cell NHLs is the extra nodal site [8, 9]. This study showed that the majority of NHLs cases were noticed among men with male: female (M: F) ratio of 1.6: 1. This finding was in agreement with previous study conducted by Economophos T and his colleagues, whom they found M: F ratio of 1.2:1. This result also was granted by Anubi CC and his colleagues whom they found that M: F ratio was 1.8: 1. [11, 21]. Nancy L. Eby and her colleagues confirmed the result of our study; they noted slightly higher incidence among men, while Salwa H. S. study in Soba University Hospital during the period 1979-1989 reported highest incidence of NHLs among men with (M: F) ratio 4.1: 1. Although her findings were much higher than other studies, they still support this study [6, 17]. The distribution of NHLs in this study according to the age ranged between 5 years to 87 years. The same result was obtained by Salwa H. S. She noted that the age of cases was ranged (0-9) years. Also Carolyn freeman et al coincides with the study result; they found that the age of patients with brain NHLs was ranged (9-80) years. Slightly different result obtained by Abdurrahman Isikdogan et al in Turkey; they reported that the age ranged from 14 years to 90 years [6, 12, 16]. In this study the age group (47-67) years accounted the highest frequency of NHLs (38.7%). This result was comparable to previous result obtained by Anubi A et al which showed that 65% of NHLs were among age group (46-55) years. Also Ameen R. et al found that most cases were within age group (41-60) years [21, 22]. The mean age in this study was 47 years. This result was equivalent with no marked difference with the findings obtained by S. C Sarel et al; they found that the mean age was 43.2 years. Abdurrahman Isikogan et al they reported 43 years as mean age. Ameen R et al found slightly higher result. Their mean age was 49 years [14, 16]. The relationship between the site of biopsy and NHLs in this study revealed that the nodal NHLs constituents 40.3% followed by stomach (19.4%) and then small and large intestine (8%). S. C Sarel et al in Turkey reported that more than half of cases (54%) were nodal NHLs [14]. The extra nodal NHLs constitute 59.7% in our study. This result is related to Ameen R et al; they noticed that 54% of the cases were extra nodal presentation. Abdurrahman Isikogan et al reported slightly different result. They found extra nodal involvement in 44.5% of patients [14, 16]. The distribution of the site of biopsy among the lymph node locations in this study revealed that the cervical lymph node was representing the higher frequency (14.5%). This result was settled with Anubi CC et al whom they found that most frequently involved lymph node was cervical lymph node [21].

## Conclusion

The study concluded the fundamental role of Immunohistochemistry stain in diagnosis and classification of NHLs into B cell and T cell. Also, the B cell NHLs constitutes the predominant immunophenotype. In addition, the age group (47-67) years was the most affected group with NHLs, and the lymph nodes were the most common site of biopsy affected by B cell NHLs. Further, extranodal locations were the most frequent sites of biopsy affected with T cell NHLs.
